# Multi-Scale Modeling for Predicting the Stiffness and Strength of Hollow-Structured Metal Foams with Structural Hierarchy

**DOI:** 10.3390/ma11030380

**Published:** 2018-03-05

**Authors:** Yong Yi, Xiaoyang Zheng, Zhibing Fu, Chaoyang Wang, Xibin Xu, Xiulan Tan

**Affiliations:** 1 School of Materials Science and Engineering, Southwest University of Science and Technology, Mianyang 621010, China; xyzheng1995@gmail.com; 2 Research Center of Laser Fusion, China Academy of Engineering Physics, Mianyang 621900, China; fuzhibingcn@163.com (Z.F.); dodolong@csu.edu.cn (X.X.); tx725@tom.com (X.T.)

**Keywords:** structure-property relations, metal foams, mechanical behavior, finite volume method, nanoporous metals

## Abstract

This work was inspired by previous experiments which managed to establish an optimal template-dealloying route to prepare ultralow density metal foams. In this study, we propose a new analytical–numerical model of hollow-structured metal foams with structural hierarchy to predict its stiffness and strength. The two-level model comprises a main backbone and a secondary nanoporous structure. The main backbone is composed of hollow sphere-packing architecture, while the secondary one is constructed of a bicontinuous nanoporous network proposed to describe the nanoscale interactions in the shell. Firstly, two nanoporous models with different geometries are generated by Voronoi tessellation, then the scaling laws of the mechanical properties are determined as a function of relative density by finite volume simulation. Furthermore, the scaling laws are applied to identify the uniaxial compression behavior of metal foams. It is shown that the thickness and relative density highly influence the Young’s modulus and yield strength, and vacancy defect determines the foams being self-supported. The present study provides not only new insights into the mechanical behaviors of both nanoporous metals and metal foams, but also a practical guide for their fabrication and application.

## 1. Introduction

Low-density metal foams have been of great interest in the past decade since the subtle combination of the property features of metals and porous structure [1,2,3]. In particular, metal foams with structural hierarchy present promising application prospects in various fields, such as supercapacitor [4], catalytic [5], sensors [6,7], controlled drug release [8] and tissue scaffold [9], benefitting from the merits of high porosity, high surface area, ultralow density, high conductivity, biocompatibility, and outstanding mechanical Performance [10]. Moreover, synthetic hierarchically porous materials constructed by natural systems could be used to introduce advantageous features in terms of performance and sustainability. The main advantageous features of hierarchically porous materials are numerous synthesis approaches, tunable porous structures, controllable macroscopic morphologies, adjustable multiple functions, and potential wide utilization [11,12]. One crucial motivation for the advancement of metal foams is the requirement in high energy-density laser experiments. Ultralow density high Z (i.e., high atomic number, high density and high radiation opacity) metal foams are desirable as the hohlraum walls in inertial confined fusion (ICF) to diminish energy dissipation and elevate energy transfer efficiency. Latest studies have demonstrated that hohlraum walls made of lower density foamed counterparts result in much lower X-ray energy loss and higher radiation temperature [13,14,15]. Therefore, a systematic strategy to design and fabricate ultralow density high Z metal foams is significant to meet the demands of ICF facilities.

**Figure 1:**
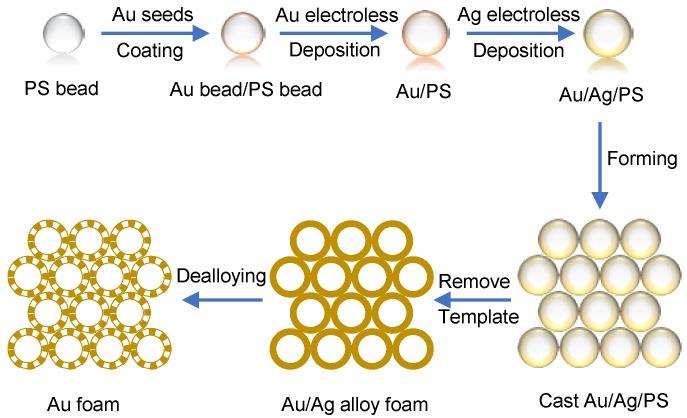
A scheme of the template-dealloying process for synthesizing Au foam.

**Figure 2:**
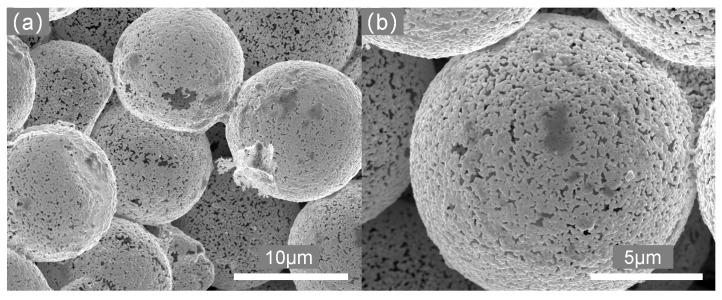
Structural characterization of hollow Au foam: main backbone consisted of hollow sphere-packing model (**a**) and bicontinuous network in the shell (**b**).

Over the last decade, a great number of synthesis strategies have been developed to fabricate hierarchically porous materials, such as dual surfactant templating, colloidal crystal templating, polymer templating, bioinspiring process, and flame transport approach [1,2,3,16,17]. This work was inspired by previous experiments which were managed to establish an optimal template-dealloying route to prepare ultralow density Au foams [18,19,20]. The detailed template-dealloying process to fabricate hollow-structured Au foams with structural hierarchy was revealed in our previous research [19,20], and a schematic illustration is shown in Figure 1. Polystyrene (PS) microspheres with diameter of 1∼10 μm, ${\mathrm{HAuCl}}_{4}$ and ${\mathrm{AgNO}}_{3}$ were adopted as the sacrificial template, Au and Ag sources. After electroless deposition of Au and Ag onto PS beads, self-supported Au–Ag bimetal foams with hollow-shell morphology were prepared by filter-cast forming of PS/Au/Ag microspheres and heat-treatment of the preformed monolithic samples. Self-supported Au foams with bimodal porous structure were produced after dealloying of Ag. Figure 2 presents the microstructure of prepared Au foam which exhibits the unique features of the combined large void space in the core and the bicontinuous nanoporous gold (np-Au) network in the shell.

Understanding the stiffness and strength of such metal foam under uniaxial compression could provide insights for their both synthesis and applications under extreme conditions. The above-mentioned metal foam consists of a two-level model: the first one is bicontinuous nanoporous model proposed to describe the nanoscale interactions in the shell, another is hollow sphere-packing model representing the micrometre-scale geometrical structure of bulk foam. A common computational simulation method to investigate the mechanical properties of foams is finite elements method (FEM) [21,22,23,24,25,26,27,28], in which the geometries are generated from nanoscale/microscale resolution X-ray computed tomography (nano-CT or micro-CT) [21,22], focused ion beam scanning electron microscopes (FIB-SEM) [23,24], phase field method [28], and some of modeling software [25,26,27,28]. Moreover, molecular dynamics also is a powerful tool to describe the movements of atoms or molecules in a large system to obtain the physical properties for nanoporous metals [29,30,31]. In this work, however, another method in terms of finite volume method (FVM) was used to investigate mechanical properties of the new topological materials through simulation and fine-tune the design. The advantages of FVM are available usage on unstructured grids, integral formulation of conservation laws, and basis on cell averaged values, which means more effective and fast compared to conventional methods [32,33,34,35].

**Figure 3:**
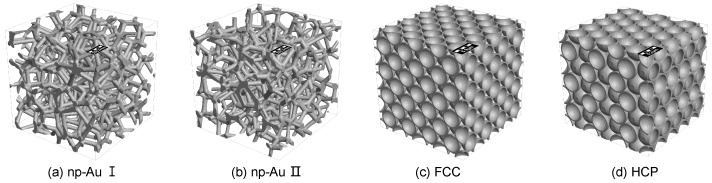
Renderings of the np-Au network (**a**,**b**) and sphere-packing Au foams (**c**,**d**).

In this study, we propose a two-level model for metal foams. The main backbone is assumed as hollow sphere-packing model (i.e., Au foam, see Figure 3c,d), while the secondary bicontinuous nanoporous network (i.e., np-Au, see Figure 3a,b) represented the interactions in the shell is modelled by Voronoi tessellation. Then, the mechanical properties of np-Au and Au foams are investigated according to finite volume method. The scaling laws for the Young’s modulus, yield strength and Poisson’s ratio of np-Au derived from the simulation are then applied to identify the uniaxial compression behavior of metal foams. Based on this approach, the other effects (e.g., shell thickness and vacancy defect) are studied to predict the stiffness and strength of Au foams.

## 2. Simulation Methods

### 2.1. Modeling

Micrographs of the shell (Figure 2b) exhibit a three-dimensional bicontinuous interpenetrating ligament (solid)–channel (void) structure, and the overall and interior morphologies of synthesized foams are shown in our pervious papers [19,20]. The network structure of the shell is generated by dealloying process, recent studies have demonstrated that np-Au fabricated by dealloying is an isotropic solid with bicontinuous structure according to 3D reconstitution by FIB-SEM [24,28]. Hence, we used the Voronoi tessellation to generate the similar structure to investigate the mechanical properties of the shell (which was considered as np-Au). All of the models of np-Au with structurally isotropic were generated by using GeoDict [36], and the detailed modeling approach is introduced in [37,38]. The main advantage of the modeling method is that the straightforward controllability on parameters such as volume fraction and geometry. The np-Au network is generated internally based on a random homogeneous pack of spheres called Basis Pore-Geometry, whose diameter determines the size and volume fraction of np-Au. Furthermore, it is assumed that the ligaments are cylindrical with diameter of 50 nm, which corresponds to prefabricated Au foams. Based on the continuum mechanics theory, although, the dimensions of the structures relative to the average ligament size used in FEM/FVM computations are found to be free from “size effects” or “surface effect” due to stress-free specimen boundaries [39,40]. Previous study has shown that the pore size and ligament diameter of np-Au are subject to Gaussian distributions [24]. In this study, hence, two different models are used to simulate the mechanical behavior: one has a constant distribution of pore size and ligament diameter, and is represented as ‘np-Au I’; the other has a Gaussian distribution of pore size and ligament diameter, and is represented as ‘np-Au II’. Table 1 summarizes the geometrical properties of the two kinds with different relative density (i.e., volume fraction of the solid phase, $\rho =\rho_{solid}/\rho_{bulk}$). The complete 3-D model of the representative volume element (RVE) consists of 4×4×4 unit cells with random realization, which is found sufficient with respect to accuracy and computation time (see Figure 3a,b).

To future predict the extreme stiffness and strength of hollow-structured Au foams, two sphere-packing models (see Figure 3c,d), namely face centered cubic (FCC) and hexagonal close packing (HCP), are generated with the same radius of 2.5 μm (r). The FCC and the HCP packing density value is the highest theoretically possible value for any sphere-packing lattice. The shell thickness (t) is set to range from 0.2 to 1 μm. The complete 3-D model of the RVE consists of 4×4×4 unit cells for FCC and 4×3×3 unit cells for HCP (here, the RVE is without randomization and theoretically only single domain can fulfil the task, but the size is particularly chosen to investigate the effect of vacancy defect). The relative density of the two foams can be calculated by:

(1) $$\rho =\frac{\sqrt{2})}{6}\pi \frac{r^{3}-{\left(r-t\right)}^{3}}{r^{3}}$$

### 2.2. Finite Volume Simulation

Finite volume simulations were performed using a commercial software GeoDict (Ver. 2017, Math2Market GmbH, Kaiserslautern, Germany) [36]. All of the models used in simulation were with size around 300×300×300 voxels, which is found sufficient with respect to accuracy and computation time [33]. The stiffness and strength for macroscopic problem can be obtained by computing the linear elastic properties and nonlinear large deformations of the structures, respectively. Thus, the module ElastoDict-VOL and ElastoDict-LD were carried out to calculate stiffness (i.e., Poisson’s ratio and Young’s modulus) and strength (i.e., yield strength and compression behavior) [33,34,35]. The material behavior was isotropic elasticity for the determination of the macroscopic stiffness, and ideal (isotropic) plasticity for the plastic determination of the macroscopic strength.

For the computation of effective stiffness, the load case was set to 0.005 uniaxial compressive strain increase along the *z*-axis, assuming a small deformation. For the computation of effective strength, the load case was set to 1% uniaxial compressive strain increase along the z-axis, assuming as a path-controlled small deformation. The boundary condition in tangential direction was set to free in order to avoid constraining the boundaries for the compression. Both the two computations were with periodic boundary condition and 0.0001 tolerance, and the load was applied as a homogeneous displacement of all nodes on the top side of the RVE. The overall Young’s modulus and Poisson’s ratio were calculated from an overall linear compression strain of 0.005. The yield strength was defined as the 0.2% offset stress from the nonlinear compression stress-strain curve.

Firstly, a study of the mechanical properties of np-Au was carried out with the following material parameters: $E_{lig}=79\;\mathrm{GPa},\;\nu_{lig}=0.42$. For the elastic–plastic behavior, the yield stress is set to $\sigma_{lig}=500$ MPa (isotropic plasticity, no work hardening) which is taken from [27]. The first simulation gave the mechanical parameters of np-Au which was closed to the isotropic law (previous study has suggested that np-Au is structurally isotropic [28]) Then, in order to investigate the mechanical properties of the bulk foam, the isotropic law of np-Au is set as the material parameters of the Au foam shell.

## 3. Results and Discussion

### 3.1. Mechanical Properties of Nanoporous Gold

#### 3.1.1. Compression Behavior

**Figure 4:**
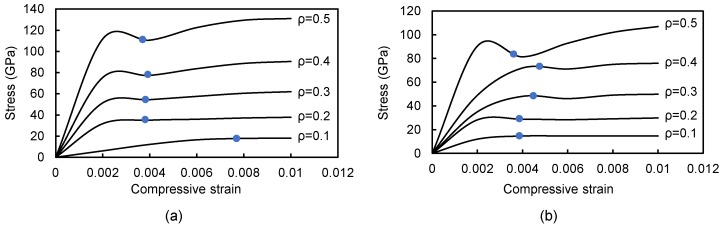
Overall compressive stress-strain curves of np-Au I (**a**) and np-Au II (**b**). The 0.2% offset stress is indicated on the curves by filled circles.

The macroscopic stress-strain curves obtained from the simulations on the np-Au samples with relative density ranging from 0.1 to 0.5 are presented in Figure 4. The relative density ρ significantly influences the deformation behavior of np-Au in stiffness, seen in the initial slope of the curves, as well as in strength, visible as the 0.2% offset stress indicated on the curves by filled circles. In addition, it should be noted that the peak stress become more distinct with increasing relative density. It is shown that the compressive behavior consists of two different deformation stages in early compression (srain <0.1). Initially, structural elements (i.e., ligaments) suffering the bending demonstrate a linear elastic compression behavior. With the further compression, the open cells collapse into plastic yielding and thus the stress-strain curve exhibits a definite plateau. Moreover, the linear elastic region for np-Au I with higher relative density ends at strain of about 0.25, while the region for np-Au II exhibits a more complicated variation due to its relative amorphous structure.

#### 3.1.2. Young’s Modulus and Yield Strength

**Figure 5:**
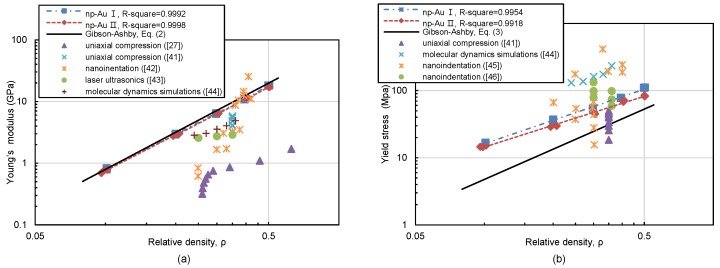
Variation of Young’s modulus (**a**) and yield stress (**b**) of nanoporous gold with relative density. Simulation results from this study for the two nanoporous gold samples are denoted as “np-Au I” and “np-Au II”. The solid line represents the Gibson and Ashby law with $E_{lig}=79$ GPa and $\sigma_{lig}=500$ MPa corresponding to bulk polycrystalline gold. Experiment results are also compared [27,41,49,50,51,52,53].

The mechanical properties are also of great interest since porous materials can have higher specific stiffness and strength relative to fully dense materials. Because of the “smaller is stronger” effect [41,42,43,44], the nanostructured porous materials with nanoscale ligaments are supposed to be stronger than conventional foam materials by up to one order of magnitude. On the other hand, by suppressing the coarsening and maintaining an unchanged network connectivity in Pt-doped np Au(Ag), Liu et al. found that the Young’s modulus varied with relative density in a power-law scaling which are free of “scale effects” [45]. In our simulation, the results are also free from “scale effects” or “surface effect” due to stress-free specimen boundaries. The classical scaling laws of Gibson-Ashby originally proposed for have been borrowed, the Young’s modulus *E* and yield strength σ for open-cell micro foams can be expressed by [46]:

(2) $$E^{*}/E_{lig}=\rho^{2}$$

(3) $$\sigma^{*}/\sigma_{lig}=0.3\rho^{3/2}$$

where $E^{*}$ and $\sigma^{*}$ are the overall foam Young’s modulus and yield strength, $E_{lig}$ and $\sigma_{lig}$ are the Young’s modulus and yield strength of ligaments materials, and ρ is the relative density. in our simulation. The relative Young’s modulus $\left(E^{*}/E_{lig}\right)$ and relative yield strength $\left(\sigma^{*}/\sigma_{lig}\right)$ of np-Au varied with relative density in a power-law relation from simulation results are now plotted in Figure 5a,b, respectively. The axis scales are log–log, so that the results along with the Gibson–Ashby’s prediction of Equations (2) and (3) show up as the straight lines. The scaling laws of relative Young’s modulus and relative yield strength for np-Au I and np-Au II can be estimated by fitting these data, respectively:

(4) $$E_{I}^{*}/E_{lig}=0.924\rho^{2.020}$$

(5) $$E_{II}^{*}/E_{lig}=0.834\rho^{1.959}$$

(6) $$\sigma_{I}^{*}/\sigma_{lig}=0.519\rho^{1.258}$$

(7) $$\sigma_{II}^{*}/\sigma_{lig}=0.370\rho^{1.136}$$

Here $E_{lig}$ = 79 GPa and $\sigma_{lig}$ = 500 MPa, which are the Young’s modulus and yield strength of ligament materials in our simulation. The firstly compression between np-Au I and np-Au II shows that the former has a slightly higher stiffness and strength than the latter, the results indicate that the pore size and ligament diameter subjected to Gaussian distributions tend to damage the mechanical properties of nanoporous metals. It is in good agreement with pervious study that determined nanoporous metals with a randomized structure tend to have a reduction of modulus and yield stress than that with a periodic structure [27]. Moreover, the scaling laws for the Young’s modulus from simulation remain significant agreement with Gibson-Ashby law. The scaling laws for the yield strength show a slightly higher order of magnitude than Gibson-Ashby’s prediction, although the exponent of the power law (1.136 and 1.258) is less than Gibson-Ashby law (3/2). In addition, pervious research demonstrate that the exponent of the power law *n* is commonly used to assess the deformation behavior of porous materials [47,48]. The exponent value for the Young’s modulus of np-Au I and np-Au II are approximately 2, which indicates a near stretching-dominated behavior.

Figure 5 also compares the results for Young’s modulus and yield stress of np-Au characterized by various experimental methods. It clearly shows that the Young’s modulus and yield stress of np-Au can vary by two orders of magnitude for a certain relative density. The main reasons come from the following three aspects. First of all, the mechanical response of np-Au depends on not only the relative density but also the topological geometry of the np structures, generally an ordered structure is stiffer and stronger than a stochastic structure [27,28]. Besides, experimental investigations were derived from np-Au with feature size ranging from several to hundreds of nanometers, and due to “size effect”, np-Au has been shown to support the “smaller is stronger” trend down to characteristic length scales as small as 10 nm [39,40,43,49]. Furthermore, the network connectivity has been suggested as a structural feature that accounts for the anomalous mechanical properties of np metals, while it tends to decrease during the coarsening in which some ligaments pinch off [44,45]. In our simulation, however, the models are free of “size effect” and assumed as open-cell foam without ligaments pinch off.

#### 3.1.3. Poisson’s Ratio

**Figure 6:**
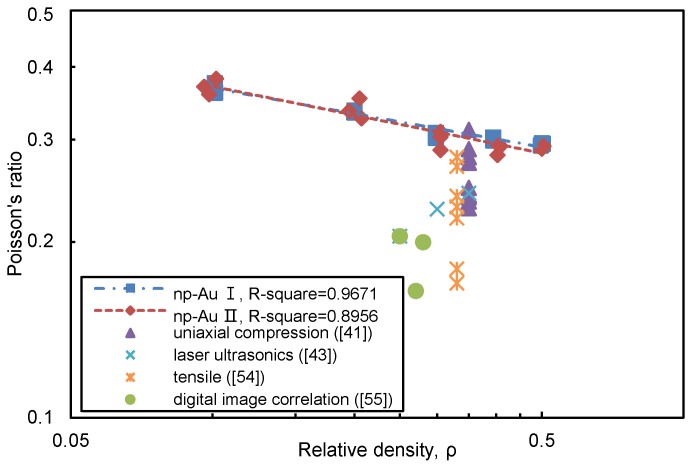
Variation of Poisson’s ratio of nanoporous gold with relative density. Simulation results from this study for the two nanoporous gold samples are denoted as “np-Au I” and “np-Au II”. Experiment results are also compared [41,50,54,55].

The elastic Poisson’s ratio of np-Au $\left(\nu^{*}\right)$ is now plotted in Figure 6 as a function of ρ. The scaling laws of $\nu^{*}$ for np-Au I and np-Au II can be estimated by fitting these data, respectively:

(8) $$\nu_{I}^{*}/\nu_{lig}=0.263\rho^{-0.146}$$

(9) $$\nu_{II}^{*}/\nu_{lig}=0.254\rho^{-0.165}$$

It shows a trend of highly similarity between the simulation results from np-Au I and np-Au II, and indicates the Poisson’s ratio is rather influenced by the structural irregularity than by the densification [40]. Furthermore, the average Poisson’s ratio values from our simulation is slightly higher than that values from experiments. Similar to the Young’s modulus and yield stress, the lower value of elastic Poisson’s ratio from experiments can be attributed to the network connectivity and ligaments size. Firstly, because these nanostructures are with less dense network connectivity resulted from pitch-offed ligaments, they could be expected to be able to accommodate a larger amount of axial deformation without expanding laterally, which could explain the lower value of Poisson’s ratio. In addition, some measurements were conducted on a specimen with smaller average ligament size and which had previously been tested and ruptured in tension [50].

### 3.2. Mechanical Properties of Gold Foam

The computational simulation results presented in Section 3.1 give a first insight into the scaling laws for Young’s modulus, yield strength and Poisson’s ratio of np-Au. In the next sect, the shell material of Au foams is assumed as bulk np-Au and the mechanical properties derived from above scaling laws of np-Au II (which has the extremely high structural similarity with np-Au from dealloying) is listed in Table 2.

#### 3.2.1. Young’s Modulus and Yield Strength

**Figure 7:**
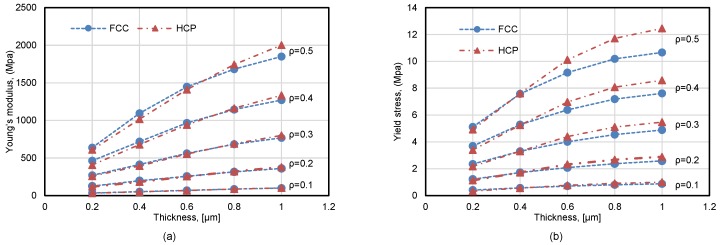
Dependence of Young’s modulus (**a**) and yield stress (**b**) of Au foams on thickness, including the variation with the type of sphere-packing and the relative density of the shell.

The Young’s modulus and yield stress of Au foams varied with the thickness t of the shell from simulation results are now plotted in Figure 7a,b, respectively. It is shown that the mechanical properties of the foams incline with the increasing thickness and/or relative density ρ of the shell. The relative density has a higher influence on the stiffness and strength than the thickness. For example, the value of Young’s modulus is about 30 MPa at *t* = 0.2 μm and ρ=0.1, and it could ascend more than 20 times to nearly 650 MPa with increasing relative density of 0.5, while the value only ascends to nearly 100 MPa with increasing thickness of 1 μm.

Moreover, the compression between FCC and HCP foams demonstrate that the type of sphere-packing models also has impact on the mechanical properties. FCC foams are stiffer than HCP foams at a low thickness value, while the difference descends gradually till *t* = ∼0.7, and then the Young’s modulus of HCP is higher than that of FCC with increasing trend. Similarly, the effects on the strength show that FCC foams have a higher yield stress while the situation changes reversedly when *t* > 0.4 μm.

#### 3.2.2. Effect of Defects

**Figure 8:**
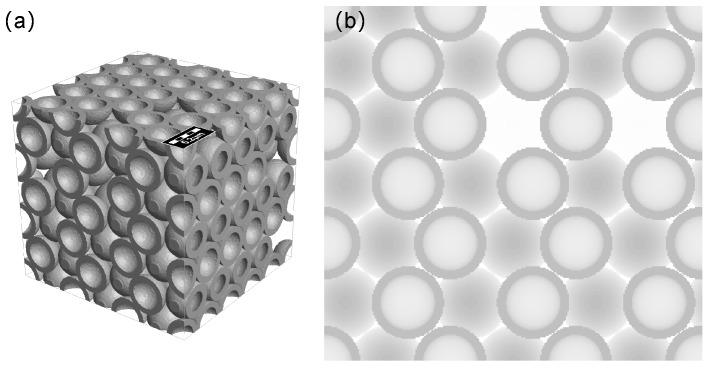
Renderings of the hexagonal close packing (HCP) foams with vacancy defect: Perspective (**a**) and top view (**b**).

**Figure 9:**
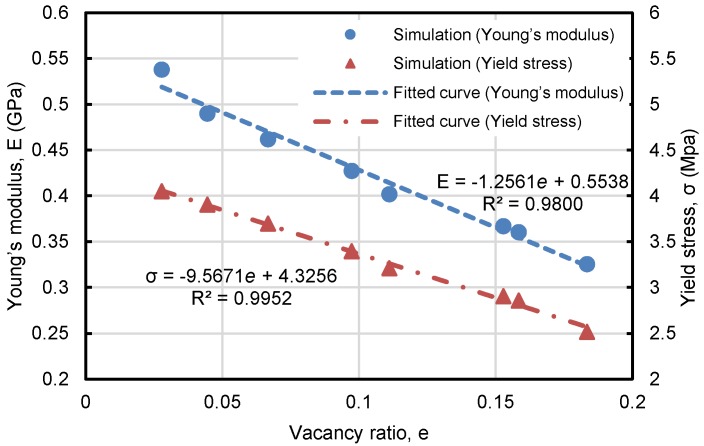
Dependence of Young’s modulus and yield stress of Au foams on vacancy ratio, including simulation results and fitted curve.

In our experiments, some hollow spheres tend to fracture resulted from some inevitable factors during the fabricating process (see Figure 2a). Most of this cracked spheres have less mechanical contribution to the bulk foam. Hence, in order to investigate the effects of such defects, we consume the cracked spheres as vacancy defect according to remove some of hollow spheres in a foam (see Figure 8). Every foam with void was generated by random removing hollow spheres according to a stochastic function, which was set to reduce the effect of the anisotropy. Vacancy content in foams represented vacancy ratio (*e*) is defined below:

(10) $$e=V_{v}/V_{s}$$

where $V_{v}$ is the volume of the vacancies (i.e., the removed spheres), and $V_{s}$ is the volume of the remaining spheres. The simulation results from a series HCP foams with t=0.6 and ρ=0.3 are shown in Figure 8. It is indicated that both the Young’s modulus and yield stress are shown a highly linear relationship with vacancy ratio e. According to the two equations fitted in Figure 9, the foam loses its stiffness by 40% and yield strength by 45% at e=0.18. When e>0.4, the material almost loses its entire stiffness and load-carrying capability, so the vacancy defect should be controlled accordingly to warrant the desired mechanical properties of metal foams.

## 4. Conclusions

In summary, the stiffness and strength of hollow-structured metal foams have been investigated using a two-level model by finite volume method. The primary model consisted of bicontinuous nanoporous structure is firstly constructed to predict the mechanical properties of the shell. A number of scaling laws have been found for the effective Young’s modulus, yield stress, and Poisson’s ratio of np-Au. Furthermore, the stiffness and strength of Au foams under uniaxial compression are carried out by combining the above scaling laws with hollow FCC and HCP. It is found that FCC is stiffer and stronger than HCP while the situation changes reversedly with the increasing thickness. Moreover, compared to the thickness, the relative density of the shell has a higher impact on the mechanical properties. Vacancy defect is one of the crucial issues influencing the foams being self-supported. Overall, in addition to predict the mechanical properties of both nanoporous metals and metal foams, we envisage that this new model can be applied in more analytical–numerical studies to reveal other interesting properties.

## Figures and Tables

**Table 1:** Geometrical Parameters of np-Au I and np-Au II.

-	Relative Density	Pore Size (nm)			Ligament Diameter (nm)		
		Mean Value	Standard Deviation	Distribution Bound	Mean Value	Standard Deviation	Distribution Bound
np-Au I	0.1	250	0	0	50	0	0
	0.2	170	0	0	50	0	0
	0.3	132	0	0	50	0	0
	0.4	112	0	0	50	0	0
	0.5	95	0	0	50	0	0
np-Au II	0.1	250	50	100	50	10	20
	0.2	170	34	68	50	10	20
	0.3	132	26.4	52.8	50	10	20
	0.4	112	22.4	44.8	50	10	20
	0.5	95	19	38	50	10	20

**Table 2:** Results from scaling laws for Young’s modulus, yield stress and Poisson’s ratio derived from Equations (5), (7) and (9) respectively.

Relative Density	Young’s Modulus (GPa)	Yield Stress (MPa)	Poisson’s Ratio
0.1	0.724	13.511	0.371
0.2	2.815	29.694	0.331
0.3	6.230	47.067	0.309
0.4	10.946	65.259	0.295
0.5	16.947	84.087	0.284
